# The use of projected autonomy in antenatal shared decision-making for periviable neonates: a qualitative study

**DOI:** 10.1186/s40748-023-00168-y

**Published:** 2023-12-01

**Authors:** Megan J. Thorvilson, Katherine Carroll, Bethany D. Kaemingk, Karen S. Schaepe, Christopher A. Collura

**Affiliations:** 1 Department of Pediatric and Adolescent Medicine, Mayo Clinic, 200 1st St SW, Rochester, MN 55905 (507)-255-0117 USA; 2 School of Sociology, College of Arts and Social Sciences, Australian National University, Canberra, Australia; 3 Division of Neonatal Medicine, Mayo Clinic, Rochester, MN USA; 4 Department of Pediatrics, Sanford Children’s Hospital, Fargo, ND USA; 5 Department of Pediatrics, University of North Dakota, Grand Forks, ND USA; 6 Robert D. and Patricia E. Kern Center for the Science of Health Care Delivery, Mayo Clinic, Rochester, MN USA

**Keywords:** Projected autonomy, Prenatal care, Decision making, Neonatologists

## Abstract

**Background:**

In this study, we assessed the communication strategies used by neonatologists in antenatal consultations which may influence decision-making when determining whether to provide resuscitation or comfort measures only in the care of periviable neonates.

**Methods:**

This study employed a qualitative study design using inductive thematic discourse analysis of ‘naturally occurring data’ in the form of antenatal conversations around resuscitation decisions at the grey zone of viability. The study occurred between February 2017 and June 2018 on a labor and delivery unit within a large Midwestern tertiary care hospital. Participants included 25 mothers who were admitted to the study hospital with anticipated delivery in the grey zone of viability and practicing neonatologists or neonatology fellows who partnered in antenatal consultation. We used a two-stage inductive analytic process to focus on how neonatologists’ discourses constructed SDM in antenatal consultations. First, we used a thematic discourse analysis to interpret the recurring patterns of meaning within the transcribed antenatal consultations, and second, we theorized the subsequent effects of these discourses on shaping the context of SDM in antenatal encounters.

**Results:**

In this qualitative study, that included discourse analysis of real-time audio conversations in 25 antenatal consults, neonatologists used language that creates projected autonomy through (i) descriptions of fetal physiology (ii) development of the fetus’s presence, and (iii) fetal role in decision-making.

**Conclusion:**

Discourse analysis of real-time audio conversations in antenatal consultations was revelatory of how various discursive patterns brought the fetus into decision-making, thus changing who is considered the key actor in SDM.

## Background

The anticipated delivery of periviable neonates necessitates supportive counseling of pregnant women by perinatal providers [1]. In the imminent delivery of a neonate at the grey zone of viability, 22 to 24 weeks of gestation, shared decision-making (SDM) among clinicians and parents is the most supported approach [1–3] to determine whether the provision of resuscitation or comfort-focused care is in the best-interest of the newborn [1–8], and challenges related to training and communication skills can compromise the encounter [9–13].

Further, SDM must account for pluralistic values and preferences among families, providers, and institutions [14–19]. As such, wide variation exists in both clinical practice and antenatal consultation of families [13, 16–18, 20] with treatment decisions for extremely preterm infants resulting in long-lasting moral distress for families and providers [21].

In SDM for periviable infants, neonatologists may play the role of choice architects, tasked with framing parental understanding of the medical decision in the context of clinical facts [11] This role requires neonatologists deliver understandable information while balancing personal beliefs to help parents express their own values towards the formulation of goals of care [22, 23]. As such, decision-making for periviable neonates is subject to significant biases, including framing effects, anchoring, optimism, or implicit prejudgment [4, 24–26]. The use of language in the counseling encounter may play a significant role in shaping understanding, constructing goals, or compelling decision-making.

In this study, we aimed to better understand the communication strategies used by neonatologists which may influence decision-making when determining whether to provide resuscitation or comfort measures only in the care of periviable neonates.

## Methods

This study employed a qualitative study design using inductive thematic discourse analysis of ‘naturally occurring data’ [27, 28] in the form of antenatal conversations around resuscitation decisions at the grey zone of viability. The objects of investigation are the transcripts derived from 25 real time audio-recorded antenatal consultations between neonatologists and pregnant women within a large Midwestern tertiary care hospital in the USA. The study was approved by Mayo Clinic’s Institutional Review Board (IRB #15-003365).

Antenatal consultations were purposively sampled and audio-recorded according to the following criteria: (1) mothers were admitted to the study hospital with anticipated delivery in the grey zone of viability defined as 22 0/7 to 24 6/7-weeks’ gestation, or when additional factors prompted SDM regarding resuscitation status for an extremely preterm neonate (ii) antenatal consultations were conducted by either practicing neonatologists or neonatology fellows (hereafter “neonatologists”). In the majority of antenatal consultations (22/25), a mother’s partner or other family member was present.

Between February 2017 and June 2018, 25 of 28 families approached by the study team consented to participate. Eligible mothers were not approached for consent in cases of precipitous labor and delivery. Neonatologists started audio-recorders upon commencing the consultation and stopped audio-recorders at the end. Participants were informed they could request the audio-recording be turned off at any time; no recording was interrupted in our study. The consults were anonymized by the removal of participant names, assigned a randomly-generated reference number, and transcribed by expert qualitative transcriptionists.

Demographic data of the participating pregnant woman including sex, race, age, marital status, pregnancy gestation, and birth outcome were collected. The participating neonatologists were represented by 2 females and 8 males; all identified as white race. One of the neonatologists both designed and participated in the study. That neonatologist removed himself during the teams’ analysis of his two consultations to allow for frank discussion by the research team. Following an interpretivist qualitative methodology, all participants and researchers were cast with active involvement in meaning construction as an interpretive practice during both the recorded antenatal conversation and our research analytic practice [29]. Details of consultations were previously published [30].

Discourse analysis unpacks the assumptions that underpin what is said, [31] and the potential consequences for those who are positioned by these discourses [32, 33]. We used a two-stage inductive analytic process to focus on how neonatologists’ discourses constructed SDM in antenatal consultations. First, we used a thematic discourse analysis to interpret the recurring patterns of meaning within the transcribed antenatal consultations, and second, we theorized the subsequent effects of these discourses on shaping the context of SDM in antenatal encounters [27, 32]. The team initially read five transcripts to discuss, determine, and agree upon the major themes in the text that were of interest to clinical practice. One researcher (KC) then returned to the remaining 20 transcripts to complete preliminary coding and develop a code book. Each co-author then reviewed the coded transcripts and code book for accuracy. Further details on the coding process and the development of themes are published elsewhere [30]. This paper examines one theme, entitled Projected Autonomy, that was deemed by the clinical researchers (CC and MT) to be clinically relevant because it was both pervasive in the data set (Table 1) and pertinent to the study focus on SDM. A second researcher (MT) conducted a discourse analysis of this theme across the 20 antenatal consultations where it was present, with a focus on the assumptions embedded in the thematically coded discourse and their effects on establishing antenatal SDM.

**Table 1:** Distribution of Discursive Strategies that Produce Projected Autonomy:

Consult No.	Fetal Physiology	Development of the Fetus’s Presence	Fetal Role in Decision Making
441		*	
180	*		
723	*		
582	*		*
332		*	
611	*		
41			*
118	*		
145		*	*
210	*		
949	*		*
824	*	*	
678	*		
339	*		
393	*		*
714	*		
866	*		
516	*		
728	*		
276	*	*	

## Results

In this paper, we focus on the common practice of producing “Projected Autonomy” in antenatal consultations. We use the term projected autonomy to describe the use of language granting the fetus the right of participation or even ownership of decision-making during antenatal consultations. Our discourse analysis revealed that neonatologists used language that creates projected autonomy in 20 of 25 consults recorded in our study, albeit with varying degrees of impact (Table 1).

Projected autonomy changed the role of the fetus from a recipient of medical interventions at birth to an actor within medical decisions in the NICU. Projected autonomy was especially produced by neonatologists when their consultations included descriptions of neonatal physiology, the anticipatory guidance of how the neonate may respond to intensive care, and the baby’s role in decision-making. To examine how projected autonomy features in antenatal consultation, we first explored three analytic categories (i) fetal physiology (ii) development of the fetus’s presence, and (iii) fetal role in decision-making.

### Fetal physiology

The first category of neonatologists’ communication contributing to projected autonomy is “fetal physiology” (Table 2) Fetal physiology was the most pervasive discursive device throughout the consults, appearing in 16 of 25 (Table 1).

**Table 2:** Categories, Definitions, and Examples of Projected Autonomy

Sub-categories	Definition	Examples
Physiology	Neonatologists describe the complexity of neonatal physiology, making it more accessible by making the fetus the subject of the actions.	*Limits* • Won’t be strong enough to do it on their own • Don’t realize they need to make the transition • Won’t be able to breathe on his own. • They don’t know how to feed on their own. *Responder* • We’ll watch and see how he’s responding… • Based on how your child’s responding • If he doesn’t respond, it’s just too early for him • We’ll see how he responds – how he’s growing, how his activity is over time *Recipient of Help* • We’ll help them breathe, stay warm, support blood pressure *Actor* • Baby’s job is to grow the placenta. • He’s pumping blood through the umbilical cord. • He’s still got room to wiggle in there. • Your baby is growing. He’s becoming more mature. • Declares it’s her turn to come into the world. • He’ll figure out how to eat. • They kind of fix themselves.
Development of the Fetus’s Presence	Neonatologists refer to the fetus in a future state, beyond the NICU course.	• We want him running around terrorizing his brother. • …keeping up with his brother and sister. • I hope he’s not too much trouble.
	Neonatologists use adjectives or nouns to depict strength and vigor.	• She’s active. • She’s strong. • He or she is the boss here. • We are her soldiers, all aligned and listening. • He’s got a will to live. • Fighter like his dad. • He’s got the highest mountain to climb.

Discussing limitations in fetal physiology due to extreme prematurity, neonatologists laid the foundation for necessary intensive interventions. One neonatologist stated that neonates “don’t realize they need to make the transition” (from in utero to ex utero) to describe the potential for pulmonary hypertension and its treatment (Consult 393; line 68). Another outlined that a neonate “won’t be able to breathe on his own” (Consult 276; line 61), setting up the rationale for intubation and ventilation.

Neonatologists discursively highlighted how they attune to baby’s physiological response to initial treatment to assess the efficacy of interventions and further inform SDM. For example, “Let’s see how things go when the baby’s born, let’s see what the data looks like as far as how he is going to respond, what his head ultrasound looks like, and make a decision if we’re doing the appropriate thing, you know, after the baby’s delivered” (Consult 866; line 513).

Further promoting the role of the neonate’s physiology in SDM, some neonatologists described their role as assistants to the baby’s physiology. “We do what we need to do to keep them [neonates] be stable – help them breathe, help them stay warm, maybe help their blood pressure” (Consult 723; line 421). This description grants the fetus a more active role in managing their own physiology. With the strongest description of the neonate’s responsibility for his/her physiology, one neonatologist shared, “…but they mostly fix themselves” (Consult 714; line 472). This description gives the fetus the primary role in their physiology, minimizing the critical role of medical staff and technologies.

### Development of the fetus’s presence

The second discursive strategy contributing to fetal projected autonomy is “Development of the Fetus’s Presence” (Table 2). During the prenatal consults, neonatologists established the presence of the fetus in two primary ways: future orientation and attribution of characteristics. The neonatologists referred to the imagined life of the fetus beyond the NICU as evidence for the efficacy of medical interventions or as a counter-balance to challenging discussions around potential morbidity and uncertainty in neonatal medicine. As an example, one neonatologist described, “…a baby starts maturing to the point that if he is born early then the medicines and the procedures that we do as physicians and nurses can make a difference and that he can survive and that he can run and play with his brothers, sisters” (Consult 276; line 28). By projecting the future, neonatologists provided a positive glimpse of the child’s life beyond the ICU and advanced the premature fetus to a phase of life that is much easier for parents to conceptualize.

Neonatologists also established the neonate’s presence through attribution of characteristics. Neonatologists used adjectives or nouns that suggested strength and resilience to describe the neonate or to mirror the parents’ descriptions of the fetus. These descriptions included “active,” “strong,” “a boss,” and often appeared near the beginning of the consult or following delivery of challenging information, such as population-based survival statistics for very premature infants and the potential for death during resuscitation. For example, shortly after describing the potential of the baby not responding to resuscitation, the neonatologist asked, “Is he a fighter like his dad?” (Consult 276; line 189). These characteristics begin to define and shape the parents’ understanding of the child they have yet to meet.

Through establishing a future orientation of the neonate and attributing character traits, neonatologists shape the neonate’s presence and developing autonomy outside the womb, establishing a being who can assume more responsibility in decisions.

### Fetal role in decision making

The third discursive strategy that enhanced the fetal role in periviable decision-making appeared in five consults (Table 3). While not the most prevalent, we argue it was the most impactful.

**Table 3:** Fetal Role in Decision-Making

*Example 1*	*Then that’s something we would come and we’d talk to you and we’d say, hey listen, this is where things are at, despite all intensive care that we can offer, your baby, you know, we can’t get things where we want, and it’s basically, that’s the sign, that’s your baby telling us that, you know, just the lungs are too premature (Consult 41; lines 929–934).*
*Example 2*	*If-if find that we are starting resuscitation and that he’s just not responding then we come to you and say “you know he’s just not responding. I think he is telling us that he wants to be with mom and dad.” And literally that’s what happens we stop what we’re doing and we bring him to you, and you can be a family for a very short period of time (Consult 145; lines 276–278).*
*Example 3*	*Because sometimes, babies tell us and, ultimately, they guide the decision-making, and they tell us my body’s not ready in this world and you’re doing everything, but I’m not responding; I’m not getting better (Consult 949; lines 454–456).*
*Example 4a*	*And the other thing I’ll tell you that, you know, sometimes I don’t, I don’t know what moms and dads necessarily feel pressure alleviated, but sometimes it’s not even you making the choice; it’s your baby (Consult 393; lines 213–214)…Sometimes after, you know, the first couple days, we have more information regarding bleeding or infection and, or his lungs are really not working for him, and families make the decision to listen to the baby and, and stop the support (Consult 393; lines 225–227).*
*Example 4b*	*Your decision, whichever it is, is the right answer, um, and that’s something we just want you to know in your heart and your head, whatever you decide is the right decision, and we’ll support you. And if your baby, he, if he tells us differently, we’ll, we’ll let you know (Consult 393; lines 417–419).*
*Example 5*	*I think you know one of the challenges of what it can feel like when I come in or when one of my colleagues comes in to talk to you is that you’re making a decision whether your child may live or die. And I, my hope is that we can somewhat reframe that question and not take away a little bit about the question of your child is gonna make the decision. He or she is the boss here. We are kind of ah their, his or her soldiers, and we’re looking to make sure we’re all aligned and, and listening to your baby. And to not feel responsible of that decision because he or she is guiding it. And it’s just us listening (Consult 582; lines 345–356).*

With this strategy, the neonatologist included the fetus not only as the individual affected by the decision, but an independent contributor in SMD. In some of the examples, the language used alluded to the fetus providing important information to the neonatologist directly, including that the baby could not survive. In others, the language delegated the sole responsibility for the decision to the fetus. To explore the fetus’s role in decision making, we examine five examples in order of increasing authority delegated to the neonate.

#### Example 1

The parents ask the neonatologist when to expect greater clarity on the severity of illness for the baby, which might prompt a transition to comfort-focused care. The neonatologist grounds the response in the delivery room during the resuscitation. The neonatologist describes that the baby may not respond to resuscitation and is therefore “telling us” that their lungs are too immature. This input from the baby would then inform a recommendation to focus on comfort (Table 3, example 1).

#### Example 2

The family articulates the importance of quality of life for their child and finds reassurance when the neonatologist describes multiple opportunities to revisit decisions throughout the treatment course. As the consult progresses, the neonatologist describes the initial resuscitation. The family asks, “Are there complications with resuscitation?” The neonatologist describes the possibility of death, prompted by the neonate’s “telling us” that he wants to have time as a family (Table 3, example 2).

#### Example 3

To frame this consult, the neonatologist sets up two possible paths forward – a comfort-focused or an intensive care plan, and then outlines the common neonatal challenges, including lung disease, necrotizing enterocolitis, intraventricular hemorrhage, and the potential for surgery. The family articulates their decision to “go with the second option where you guys would do everything.” The neonatologist responds with potential roadblocks and how those roadblocks can be the baby “telling us” that their body is not ready, thus participating in the decision (Table 3, example 3).

#### Example 4

The parent asks specifically about survival rates for preterm infants. The neonatologist goes on to describe the uncertainty inherent in neonatal medicine and introduces the concept of comfort-focused care. The grandmother then states that the choice is the parents’ alone, “No one else can make that choice for you.” The neonatologist subsequently offers that it may not be the parents’ choice and offers the alternative to “listen to” the choice of the baby (Table 3, example 4a).

As the consult progresses, the family considers the difference between starting and stopping interventions. They request specific numbers for the outcome of neonates born at 22 weeks. The neonatologist again refers to the significant uncertainty for neonates at 22 weeks gestation and shares that there is no right answer. The neonatologist then pivots and assures the family that whatever decision they make will be the right one, again referencing the baby as an actor in the decision through his “telling us” of the right decision (Table 3, example 4b).

#### Example 5

The parents ask, “For me I mean it’s just at what point we have the right to have a decision about things and at what point during the process, weeks wise, what decision do we have” (lines 13–14). The mother draws from the experience of a friend’s premature infant’s death without access to tertiary neonatal care, and how their lack of opportunity “haunts” her. Following this description, the neonatologist shifts the burden of the decision from the parents (“you’re making a decision whether your child may live or die”) to the fetus (“your child is gonna make the decision”), and clarifies that the parents’ responsibility is to listen to their child (Table 3, example 5).

## Discussion

With projected autonomy, neonatologists elevated the role of the fetus in prenatal consults, granting the fetus participation in decision-making during antenatal consultations. Projected autonomy appeared in the majority of consultations. Projected autonomy’s use was most prevalent in Fetal Physiology and most impactful in Fetal Role in Decision Making. In the context of SDM, our analysis showed neonatologists used projected autonomy to efficiently convey complicated information, orient parents to the potential challenges ahead, foster a sense of hope and trust, and guide parents to a decision around resuscitation.

Referencing Fetal Physiology allowed neonatologists to distill complex information about neonatal physiology to parents, thus equipping them with information to make informed decisions [34, 35]. Beyond descriptions of physiology, the neonatologists shared how the neonate’s physiology and response to interventions could serve as a tool for frequent re-evaluations throughout the NICU course, further informing SDM.

Neonatologists’ discursive strategy of Development of the Fetus’s Presence infused hope into the prenatal consult. As parents face the potential reality of preterm delivery and the impact for their child and family, they inevitably feel fear and despair [36]. The transition from hoping for a “normal” pregnancy to the possibility of morbidity and even death is disorienting for parents [37–39]. A description of life beyond the NICU may be one strategy that neonatologists use to offset parental anxiety, allowing a moment of decompression in the high-stakes prenatal consult [40, 41]. It may also help parents that neonatologists can envision a hopeful path forward with survival for their child, thereby building trust in the neonatal team [42–44].

Beyond hope and trust, the reference to a child’s future may produce bias towards resuscitation as the outcome of the medical decision. The description of a child’s future life assumes survival beyond the NICU, and the references to milestones (i.e., running) paint a picture of a normal or near-normal childhood. The attribution of characteristics of “strong” and “a fighter” may also favor resuscitation. These powerful life-assuming adjectives describe a vigorous (yet unknown) child and carry the potential of favoring resuscitation.

The strategy utilized by neonatologists to grant the fetus a role in SDM offers the most powerful example of projected autonomy. Projected autonomy, as seen in the examples of decision-making, may allow neonatologists to offload some of the burden of the decision around resuscitation from both the parents and neonatologist [45, 46]. By introducing a new participant in the decision – the participant most affected by the decision – the decision’s weight is distributed across the parent, the neonatologist, and now the neonate. As a result of projected autonomy in SDM in the antenatal consultation, the distribution of the burden includes the neonate and creates more space for parents to attune to the neonate as the newly realized participant in the decision [47]. Further, it may even suggest that decision-making is no longer necessary if the baby projects self-determination via grave illness that further intensive interventions may no longer be helpful.

Clinically, this transformation of dyadic SDM between the neonatologist and the parents to triadic SDM transforms what was previously a binary decision at the prenatal consult – to resuscitate or not – to one that includes a third option, namely “a trial of intervention” [48]. This transformation ultimately places the response of the infant at the center of the decision and stretches this communicative role of the infant into the NICU course.

One strength of a trial of resuscitation and the extension of the decision is the mitigation of uncertainty around outcomes for extremely premature infants [48]. The inability to accurately and consistently predict the NICU course and the developmental outcomes can be uncomfortable and unnerving for neonatologists [19, 45, 49]. As we saw above, one neonatologist postponed the decision from the prenatal consult, suggesting they return to the decision in the initial days after birth when they could truly speak to the reality of the head ultrasound, risk of infection, and pulmonary function. Waiting to gather more information after the child’s birth, neonatologists create space to move further along the spectrum from uncertainty towards certainty when the stakes are so high [50].

While we caution against applying projected autonomy broadly beyond the clinical space of SDM, our study suggests that projected autonomy onto the fetus is a distinct discursive strategy that may be commonly utilized by neonatologists. This has the effect of deferring the antenatal decision into the postnatal NICU course in order to incorporate clinical events into SDM. This discursive strategy perhaps also attests to the broader discomfort of proactively discussing care at the margins of life, including the possibility of death, particularly among babies and the young in the culture of acute-care medicine [51]. By its nature, a qualitative study design aims to understand the particulars and the meaning of naturally-occurring data derived from a specific sample of the population [28, 52]. Qualitative discourse analysis of prenatal consults offers clinical staff insight to the content and efficacy of these clinical encounters. This study was conducted at a single Midwestern regional referral center and included neonatologists from a homogenous white racial background. Therefore, the main limitation to the study is transferability to other neonatal treatment locations within the United States and worldwide [52]. Despite this limitation, this study adds a unique perspective to the medical literature through the utilization of discourse analysis of real-life medical encounters during antenatal consultations.

## Conclusion

Discourse analysis of real-time audio conversations in antenatal consultations was revelatory of how various discursive patterns brought the fetus into decision-making, thus changing who is considered the key actor in SDM.

## Data Availability

Data will be kept confidential to protect the confidentiality of the patients in the study.

## Abbreviations

SDM: Shared decision making
NICU: Neonatal intensive care unit
